# Unraveling Improvisational Behavior Among Critical Care Nurses in Relation to Integral Leadership, Job Crafting, and Digital Competence: A Moderated Mediation Analysis

**DOI:** 10.1155/jonm/9316513

**Published:** 2026-11-06

**Authors:** Ahmed Abdelwahab Ibrahim El-Sayed, Ibrahim Alasqah, Boshra Mostafa Younes, Maha Gamal Ramadan Asal, Nada Alqarawi

**Affiliations:** ^1^ Nursing Administration Department Faculty of Nursing Alexandria University Alexandria Egypt alexu.edu.eg; ^2^ Department of Community, Psychiatric, and Mental Health Nursing College of Nursing Qassim University Buraydah 52571 Saudi Arabia qu.edu.sa; ^3^ Nursing Services Department Qassim University Medical City Buraydah 52571 Saudi Arabia; ^4^ Nursing Administration Department Faculty of Nursing Aswan University Aswan Egypt aswu.edu.eg; ^5^ Medical Surgical Nursing Department Faculty of Nursing Alexandria University Alexandria Egypt alexu.edu.eg

**Keywords:** clinical practice, critical care nursing, digital competence, improvisational behavior, integral leadership, job crafting, nursing staff, professional competence

## Abstract

**Aim:**

To examine the direct and indirect associations of integral leadership on critical care nurses’ improvisational behavior through job crafting and to test the moderating role of digital competence.

**Background:**

Critical care nurses work in high‐acuity, technology‐intensive environments that require rapid judgment, flexible coordination, and adaptive responses when routine procedures are insufficient. However, limited evidence explains how improvisational behavior can be shaped among critical care nurses within digitally transforming healthcare systems.

**Methods:**

A cross‐sectional analytical correlational design was used. Data were collected over three months in 2025 from 425 critical care nurses working in 31 intensive care units across 10 hospitals in Egypt. Anonymous, self‐administered questionnaires were used to assess integral leadership, job crafting, digital competence, and improvisational behavior. Data were analyzed using descriptive statistics, correlation analysis, and regression‐based moderated mediation analysis.

**Results:**

Integral leadership was positively associated with critical care nurses’ improvisational behavior and job crafting, while job crafting was positively associated with improvisational behavior. Integral leadership demonstrated a significant direct association with improvisational behavior, and its indirect association through job crafting was also significant. Digital competence was positively associated with job crafting and significantly moderated the relationship between integral leadership and job crafting, with a stronger association observed among nurses with higher digital competence. The conditional indirect association of integral leadership with improvisational behavior through job crafting was also stronger at higher levels of digital competence.

**Conclusion:**

The findings highlight the importance of considering integral leadership, proactive work redesign, and digital competence together when understanding improvisational behavior in critical care nursing. This integrated perspective may help nursing organizations create work environments that enable nurses to respond adaptively to unexpected clinical demands while maintaining safe and professionally appropriate practice.

**Implications for Nursing Management:**

Nurse managers should create conditions that support safe improvisational behavior by providing nurses with leadership support and appropriate autonomy, encouraging responsible job crafting within professional boundaries, and providing targeted digital competence training. Simulation‐based training and structured debriefing of unexpected clinical situations may also help nurses develop and reflect on adaptive responses while maintaining patient‐safety standards.

## 1. Introduction

Critical care requires nurses to respond to rapidly changing patient conditions within complex, technology‐intensive environments [1, 2]. Although standardized procedures provide an essential foundation for safe practice, unexpected situations may arise in which established routines or immediately available resources do not fully address an emerging clinical demand [3, 4]. In such circumstances, nurses may need to improvise by drawing on available knowledge, resources, and clinical judgment to generate a spontaneous and functional response [5, 6]. Improvisational behavior may therefore be particularly relevant to critical care, where responses must be both timely and clinically appropriate [7].

The conditions under which nurses can improvise may depend partly on their work environment [8]. Integral leadership may be important because its holistic orientation encompasses individual development, relationships, organizational culture, and organizational functioning [9, 10]. Yet, leadership support may not be sufficient by itself to explain improvisational behavior, because nurses may also need to proactively reshape how they perform and organize their work [8, 11]. Job crafting provides a potential behavioral pathway for this process because nurses can modify tasks, relationships, and perceptions of work to improve alignment between work demands, available resources, and professional goals [12, 13]. Thus, the present study positions job crafting as a pathway through which leadership resources may be translated into nurses′ capacity for effective improvisational responses.

This mechanism may become increasingly important as critical care becomes more digitally mediated [1, 2]. Digital competence may influence nurses′ ability to use digital information, communication systems, and clinical technologies when modifying work processes and mobilizing available resources [14, 15]. On this basis, integral leadership may create an enabling context for job crafting, while digital competence may influence how effectively nurses can convert that context into proactive work redesign [15, 16].

Although previous research has linked leadership and job crafting with innovative work behavior among nurses [17, 18], these findings primarily establish a relationship between leadership, proactive work redesign, and innovation‐oriented outcomes; they do not necessarily demonstrate that the same pathway operates when nurses must respond spontaneously to unfolding clinical demands. This distinction identifies an important unresolved issue: whether the proactive changes associated with job crafting can translate into real‐time improvisational behavior when established routines are insufficient. Addressing this question is particularly relevant in technology‐intensive critical care settings, where nurses may need to draw on existing resources and modify how they organize and perform their work while responding to rapidly changing conditions.

Accordingly, examining improvisational behavior rather than innovation‐oriented outcomes allows the present study to test whether the leadership–job crafting pathway extends from proactive work redesign to spontaneous clinical responses. It is also unclear whether digital competence strengthens the relationship between integral leadership and job crafting, providing an additional boundary condition for this proposed pathway. The present study therefore examines whether integral leadership is associated with critical care nurses’ improvisational behavior through job crafting and whether digital competence moderates the relationship between integral leadership and job crafting, thereby conditioning the indirect association with improvisational behavior.

## 2. Background and Theoretical Framework

The job demands–resources (JD‐R) model provides the overarching theoretical foundation for the present study. The model proposes that employees′ work behavior is shaped by the resources available within their work environment and by the demands they encounter, with resources such as autonomy, social support, participation, feedback, and developmental opportunities supporting employees′ capacity to manage demanding work and engage in proactive behavior [16]. This perspective is particularly relevant to critical care nursing, where high clinical demands coexist with needs for timely information, technological support, coordination, and effective clinical decision‐making [1, 2]. Within this framework, leadership can function as an important contextual resource by creating conditions that enable nurses to mobilize available resources and respond proactively to complex work demands [16].

Integral leadership provides a particularly relevant example of such a contextual resource [19]. Although integral leadership shares characteristics with empowering, inclusive, supportive, relational, and ambidextrous leadership approaches, its distinctive feature lies in its broader integrative and multilevel orientation rather than in any single leadership behavior. Integral leadership incorporates individual leadership qualities, individual performance, organizational culture, and organizational excellence, thereby connecting individual, relational, and organizational dimensions within a unified leadership perspective [9, 10, 20, 21].

In contrast, empowering leadership primarily emphasizes autonomy, participation, influence, and discretion, whereas inclusive and supportive leadership approaches focus more strongly on accessibility, interpersonal support, inclusion, and relational exchange. Ambidextrous leadership emphasizes the leader’s ability to balance exploration and exploitation, allowing organizations to pursue change and innovation while maintaining existing practices [22]. Although these approaches may overlap with integral leadership, the latter places greater emphasis on how individual, relational, cultural, and organizational dimensions interact as part of a broader work system [20]. Its incremental theoretical value therefore lies not in a unique set of leadership behaviors, but in providing a multilevel framework for understanding how these interconnected dimensions may jointly shape nurses’ responses to complex and unexpected clinical demands [19].

This broader orientation is particularly relevant to critical care nursing, where improvisational responses may be shaped by the interaction of professional judgment, team relationships, technological resources, organizational processes, and competing clinical priorities [1, 2]. When established routines are insufficient, nurses may need to coordinate these resources and conditions simultaneously to develop timely and appropriate responses [6]. Integral leadership therefore provides a theoretically relevant context for examining improvisational behavior as a response embedded within a complex clinical system, rather than as an outcome of leadership support or individual autonomy alone [10]. Accordingly, integral leadership is not proposed as inherently superior to other leadership approaches; its theoretical contribution lies in offering a broader systems‐oriented perspective for examining how leadership conditions may facilitate responsible improvisation in critical care.

Improvisational behavior refers to spontaneous, creative, and functional action in response to unexpected situations or circumstances in which established routines do not provide an adequate response [23, 24]. The construct incorporates spontaneity, creativity, and functionality, meaning that the response emerges in the moment, involves some degree of novel recombination of available resources or actions, and remains directed toward an effective outcome [25, 26]. This distinguishes improvisational behavior from broader adaptive behavior, which encompasses adjustment to changing circumstances but does not necessarily involve spontaneous creation of a response [27].

Improvisational behavior also differs from innovative work behavior, which emphasizes the generation, promotion, and implementation of new ideas [22]. Although both constructs may involve novelty and creativity, they differ in their temporal orientation and immediate behavioral purpose. Innovative work behavior generally involves developing, promoting, or implementing ideas that may extend beyond the immediate situation, whereas improvisational behavior involves constructing or adapting a response in real time when an unexpected situation unfolds and established routines are insufficient [22, 26]. Thus, a nurse may engage in innovative work behavior without improvising in an acute situation and may improvise effectively without generating an innovation intended for broader or future implementation.

In the present study, improvisational behavior is conceptualized as responsible real‐time adaptation within the boundaries of clinical judgment, professional standards, teamwork, and patient safety, rather than unrestricted departure from established procedures [4, 6]. Similarly, improvisation is not equated with informal workarounds; workarounds generally involve bypassing, modifying, or circumventing established procedures, systems, or constraints to accomplish a task, whereas the present conceptualization focuses specifically on spontaneous responses to unexpected demands that remain directed toward effective and professionally appropriate action [23].

The demanding and unpredictable nature of intensive care makes such behavior particularly relevant. Sudden patient deterioration, technology‐related problems, competing priorities, and resource limitations may require nurses to adjust their actions rapidly [1, 5]. However, improvisation is unlikely to depend solely on an individual nurse’s disposition. Leadership may shape whether nurses have the autonomy, support, information, and relational conditions necessary to respond constructively to unexpected demands [8, 28]. Integral leadership, through its emphasis on participation, shared purpose, relational collaboration, adaptability, and organizational awareness, may therefore encourage nurses to engage in responsible improvisation when routine responses are insufficient [9, 20]. Accordingly, the following hypothesis is proposed:

• H1: Integral leadership is positively associated with critical care nurses′ improvisational behavior.

Integral leadership may also influence nurses′ proactive engagement with their work by creating conditions that support employees′ efforts to modify how work is organized and performed [29]. This proactive work redesign is captured by the concept of job crafting, which refers to employees′ modification of tasks, relationships, and perceptions of work to improve the fit between work demands, available resources, and personal or professional goals [13]. In nursing, job crafting may involve modifying work structures, strengthening professional relationships, seeking and communicating information, assigning meaning to work, and maintaining a constructive orientation toward change [12]. From a JD‐R perspective, job crafting represents an active process through which employees mobilize or reshape aspects of their work environment in response to job demands and available resources [16, 30].

Within this framework, leadership can provide contextual resources that make proactive work redesign more feasible. Leaders who encourage participation, provide support, recognize professional expertise, and allow appropriate autonomy may give nurses greater opportunity to modify how they organize and perform their work [16, 29]. Recent evidence similarly links leadership with job crafting and innovative work behavior, including research involving servant leadership and job crafting among nurses [17], transformational leadership and job crafting among nurses [18], and inclusive leadership and innovative work behavior among faculty in higher education [31].

Together, this literature supports the relevance of leadership as a contextual resource for proactive work redesign, while leaving open whether this process extends specifically to improvisational behavior in critical care nursing. Given this integrative orientation, integral leadership provides a theoretically relevant leadership context for examining this relationship [19]. Based on this theoretical and empirical rationale, the following hypothesis is proposed:

• H2: Integral leadership is positively associated with critical care nurses′ job crafting.

Job crafting may, in turn, provide a behavioral foundation for improvisational behavior. By modifying tasks and work processes, nurses may become more familiar with alternative ways of organizing their responsibilities; by reshaping relationships, they may strengthen access to colleagues, information, and social support; and by reframing aspects of their work, they may develop alternative perspectives for approaching demanding situations [32–34]. These changes do not constitute improvisational behavior themselves. Rather, they may expand the repertoire of resources and possible courses of action available to nurses when established routines are insufficient [35, 36]. Existing nursing research provides support for the broader value of job crafting, linking it with outcomes such as engagement, resilience, evidence‐based practice competency, and innovative work behavior [17, 33, 37]. However, whether job crafting is associated with improvisational behavior remains unclear. Examining this relationship therefore extends the existing job crafting literature to a distinct downstream behavioral outcome involving spontaneous responses to unexpected demands. Therefore, the following hypothesis is proposed:

• H3: Job crafting is positively associated with critical care nurses′ improvisational behavior.

Digital competence may also support job crafting by enabling nurses to use technology‐related resources to modify how their work is organized and performed. It encompasses the knowledge, skills, and attitudes required to use digital technologies effectively [15]. In technology‐intensive clinical environments, digitally competent nurses may be better able to access and interpret information, use digital communication and clinical systems, and identify opportunities to improve workflows [2, 14]. Such capabilities may enable nurses to recognize opportunities for proactive changes in tasks and work processes and to mobilize digital resources when making those changes [38]. Evidence has also linked digital competence with nurses′ clinical judgment, professional well‐being, and career sustainability [39, 40], supporting its relevance as an individual capability for proactive work redesign. Accordingly, it is hypothesized that

• H4: Digital competence is positively associated with critical care nurses′ job crafting.

Beyond its direct association with job crafting, digital competence may influence how effectively leadership support is translated into proactive work redesign. Integral leadership may provide nurses with autonomy, encouragement, participation, and legitimacy to improve their work, but the extent to which these contextual resources are converted into job crafting may depend partly on nurses′ ability to use the digital resources embedded in contemporary clinical practice [20, 29]. Nurses with stronger digital competence may be better positioned to use digital information and technology‐supported resources when modifying tasks, communication, and workflows [2, 14].

Thus, digital competence may strengthen the positive association between integral leadership and job crafting by enabling nurses to convert leadership‐provided opportunities and support into technology‐enabled changes in their work. This proposition is consistent with the JD‐R model, in which the behavioral value of contextual resources may depend partly on employees′ capabilities to mobilize those resources [16]. Accordingly, it is hypothesized that

• H5: Digital competence moderates the relationship between integral leadership and job crafting such that the relationship is stronger among critical care nurses with higher digital competence.

The preceding relationships suggest that job crafting may explain how integral leadership becomes associated with improvisational behavior. Leadership may provide contextual resources and legitimacy for proactive adaptation, while job crafting represents the behavioral process through which nurses translate these resources into changes in tasks, relationships, and work processes. Such changes may strengthen resourcefulness and situational readiness, facilitating effective responses when established routines are insufficient [33, 36]. This proposed pathway extends prior leadership–job crafting research by examining improvisational rather than innovative work behavior as the downstream outcome [17, 18]. Taking together, these arguments indicate that job crafting may function as a pathway linking integral leadership with improvisational behavior. Accordingly, the following hypothesis is proposed:

• H6: Job crafting mediates the relationship between integral leadership and critical care nurses′ improvisational behavior.

Because digital competence is proposed to strengthen the first stage of the pathway, the indirect association between integral leadership and improvisational behavior through job crafting is also expected to vary according to nurses′ level of digital competence. Nurses with higher digital competence may be better positioned to translate leadership‐provided resources into proactive changes in tasks, relationships, and work processes, thereby strengthening the pathway through which job crafting may support improvisational responses. Thus, the proposed model integrates a contextual resource, a proactive behavioral mechanism, and an individual capability within the JD‐R framework: Integral leadership represents the contextual resource, job crafting represents the behavioral pathway, and digital competence represents the boundary condition of this process. Accordingly, the preceding arguments lead to the following moderated mediation hypothesis:

• H7: The indirect association of integral leadership on improvisational behavior through job crafting is conditional on digital competence such that the indirect association is stronger among critical care nurses with higher digital competence.

The proposed conceptual model integrating integral leadership, job crafting, digital competence, and improvisational behavior is presented in Figure 1.

**FIGURE 1:**
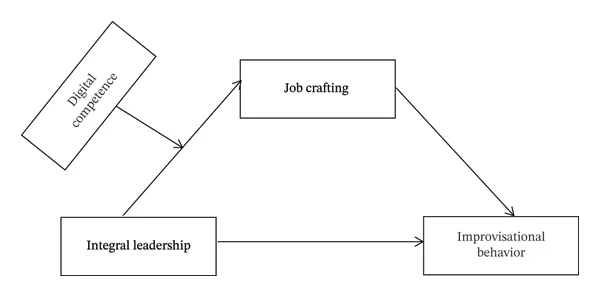
Conceptual model of the study.

## 3. Objectives of the Study

This study aimed to examine the direct and indirect associations of integral leadership with critical care nurses’ improvisational behavior through job crafting and to investigate whether digital competence moderates the association between integral leadership and job crafting.

## 4. Method

### 4.1. Design and Setting

This study employed a cross‐sectional analytical correlational design to examine the direct, indirect, and moderating relationships among the study variables at a single point in time within natural clinical settings. The design also supported testing of a theory‐driven moderated mediation model using regression‐based analytical procedures. The study was conducted in 31 intensive care units (ICUs) across 10 hospitals in Egypt. These hospitals were distributed across two governorates: seven hospitals in Alexandria, comprising 23 ICUs, and three hospitals in Aswan, comprising eight ICUs. The 10 hospitals were purposively selected to represent the major healthcare sectors in Egypt, including university hospitals, Ministry of Health and Population hospitals, Health Insurance Organization hospitals, hospitals affiliated with the General Secretariat of Specialized Medical Centers, and private hospitals. Selection was also guided by the presence of established and routinely used digital clinical systems, consistent with the study aim. Because digital clinical systems were implemented at the hospital level, all ICUs within each selected hospital were included in the study (see Figure 2). The study was reported in accordance with the Strengthening the Reporting of Observational Studies in Epidemiology (STROBE) guidelines [41].

**FIGURE 2:**
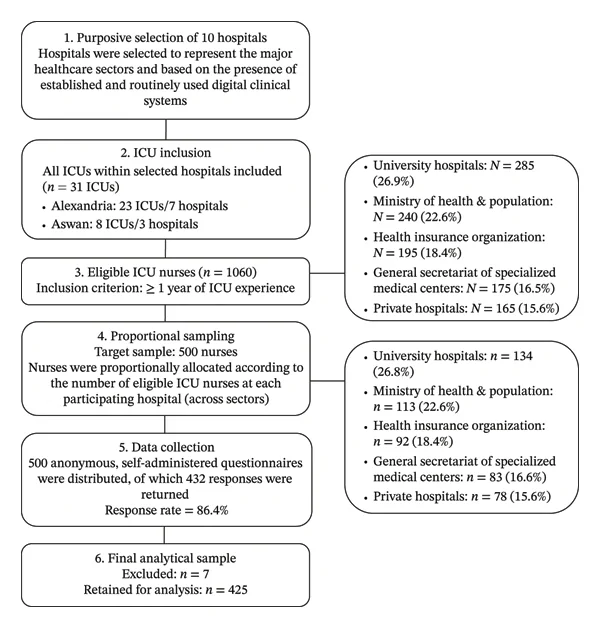
Flow of the study.

### 4.2. Participant and Sampling

The sampling frame comprised 1060 eligible ICU nurses across the 31 participating ICUs. A multistage sampling approach was used, involving purposive selection of hospitals, inclusion of all ICUs within the selected hospitals, identification of eligible nurses, and proportional allocation of the target sample across healthcare sectors (see Figure 2). The required sample size was determined using G∗Power software, Version 3.1.9.7. An a priori power analysis was conducted for the proposed moderated mediation model (PROCESS Macro Model 7), assuming a small effect size (*f* ^2^ = 0.02), a significance level of 0.05, and a statistical power of 80%. Based on Cohen’s recommendations for multiple regression analysis, the minimum sample size required to adequately test the study hypotheses was estimated at 395 participants [42]. To account for potential nonresponse and incomplete questionnaires, the target sample size was increased to 500 nurses.

### 4.3. Study Instruments

Data were collected using four standardized self‐administered instruments to assess the primary study constructs. Additionally, participants’ demographic and professional characteristics, including age, sex, educational qualification, years of clinical experience, and AI use in clinical practice, were collected. AI use was assessed using a self‐reported question asking participants whether they used AI‐enabled applications in clinical practice, with responses coded as 0 = no and 1 = yes.

Integral leadership was measured using the Integral Nursing Leadership Scale (INLS) developed by Cho and Choi (2024) [9]. Participating nurses were instructed to rate the integral leadership of their immediate supervisor or manager in their current unit; thus, the INLS assessed nurses’ perceptions of their immediate nurse manager’s integral leadership rather than their own leadership characteristics. The instrument comprises 30 items distributed across four domains: individual leadership qualities (9 items), individual performance (9 items), influencing organizational culture (7 items), and organizational excellence (5 items). Responses are rated on a 5‐point Likert scale ranging from 1 (strongly disagree) to 5 (strongly agree), with higher scores indicating higher levels of perceived integral nursing leadership. The original validation study demonstrated excellent internal consistency reliability, with Cronbach’s alpha and McDonald’s omega coefficients of 0.97 and 0.98, respectively [9].

Job crafting was assessed using the Clinical Nurses’ Job Crafting Scale (CN‐JCS) developed by Jeong and Han [12]. The scale comprises 20 items categorized into five dimensions: giving meaning to work (6 items), changing the structure of work (4 items), exploring and communicating (4 items), pursuing relationship change (3 items), and positive attitude toward change (3 items). Items are rated on a 5‐point Likert scale ranging from 1 (Not at All) to 5 (Very Much), with higher scores reflecting greater engagement in job crafting behaviors. The CN‐JCS demonstrated strong psychometric properties in the original validation study, with Cronbach’s alpha coefficients ranging from 0.73 to 0.85 across subscales and an overall reliability coefficient of 0.90 [12].

Digital competence was measured using the Digital Competence Questionnaire (DCQ) developed by Golz et al. [15]. The DCQ is a psychometrically validated instrument designed to assess digital competence among clinical nurses. The questionnaire contains 12 items encompassing two dimensions: attitude (6 items) and knowledge and skills (6 items). Responses are measured on a 5‐point Likert scale ranging from 1 (Fully Disagree) to 5 (Fully Agree), with higher scores representing higher perceived digital competence. The original scale demonstrated satisfactory psychometric properties, with Cronbach’s alpha coefficients of 0.91 for the attitude dimension and 0.81 for the knowledge and skills dimension [15].

Improvisational behavior was assessed using the Individual Improvisation Scale (IIS) developed by Hmieleski et al. [43]. The IIS consists of 12 items designed to evaluate individuals’ ability to respond spontaneously, creatively, and effectively in unpredictable situations. Responses are scored on a 5‐point Likert scale ranging from 1 (Completely Disagree) to 5 (Completely Agree), with higher scores indicating greater improvisational capability. The original instrument demonstrated good internal consistency reliability, with a reported Cronbach’s alpha coefficient of 0.84 [43].

### 4.4. Instrument Translation, Validity, and Pilot Testing

The study instruments were translated into Arabic using a standardized forward–backward translation procedure based on Sperber’s guidelines [44]. Two bilingual healthcare translators independently translated the original instruments into Arabic, after which the research team synthesized the translations into a unified preliminary version. Subsequently, two independent bilingual translators, blinded to the original instruments, performed back‐translation into English. The back‐translated versions were compared with the original instruments to ensure conceptual and semantic equivalence, and discrepancies were resolved through expert consensus.

Content validity was assessed by three professors of critical care nursing who independently evaluated the relevance of each item using a 4‐point rating scale: 1 = Not Relevant, 2 = Somewhat Relevant, 3 = Quite Relevant, and 4 = Highly Relevant. Following Polit and Beck (2020), ratings of 1 and 2 were classified as indicating insufficient relevance, whereas ratings of 3 and 4 were considered to indicate item relevance. The item‐level content validity index (I‐CVI) was calculated as the proportion of experts assigning a rating of 3 or 4 to each item, and the scale‐level CVI based on the average method (S‐CVI/Ave) was calculated as the mean of the item‐level I‐CVI values across each instrument. All items across the study instruments received ratings of 3 or 4 from all three experts. Accordingly, all items had an I‐CVI of 1.00, indicating complete expert agreement regarding item relevance, and the S‐CVI/Ave was 1.00 for each instrument.

Face validity was assessed through a pilot sample involving 50 nurses to evaluate item clarity and questionnaire completion time. Mean clarity scores ranged from 4.32 to 4.92 on a 5‐point scale, indicating high comprehensibility. The average completion time was approximately 40 min. Data obtained from the pilot study were excluded from the final analysis.

### 4.5. Data Collection Procedure

Data were collected over three months in 2025 using anonymous, self‐administered questionnaires distributed to eligible staff nurses. Following proportional allocation of the target sample across the participating hospitals, eligible nurses were approached within their respective ICUs for recruitment. Before completion, participants received standardized verbal explanations about the study objectives, procedures, eligibility criteria, voluntary participation, and estimated completion time, with opportunities for clarification. Questionnaires were completed in nonsupervisory settings during break periods to reduce workplace influence and perceived coercion. To enhance the response rate, data collectors coordinated with unit managers to identify suitable break times, allowed adequate time for completion, provided flexible return options, and made brief follow‐up visits to collect completed questionnaires. Of the 500 targeted nurses, 432 were returned, yielding a response rate of 86.4%.

### 4.6. Statistical Analysis

Data were analyzed using IBM SPSS (Version 28) Statistics and IBM SPSS AMOS (Version 22). Statistical significance was evaluated using two‐tailed tests with an alpha level of 0.05. Unless otherwise specified, 95% confidence intervals (CIs) were used.

The dataset was initially screened for questionnaire completeness. Four questionnaires with more than 5% missing responses, concentrated in the final section of the survey, were excluded because the incomplete data prevented reliable calculation of the corresponding scale score. The remaining observations were examined using standardized and studentized residuals, leverage values, Mahalanobis distance, and Cook’s distance for both regression equations underlying the moderated mediation model. Three observations (Cases 99, 243, and 365) demonstrated evidence of substantial influence across multiple diagnostic measures and were therefore excluded from the primary analyses, resulting in a final analytical sample of 425 nurses. Detailed influence diagnostics for these three observations are presented in Supporting Table S1.

Descriptive statistics, including means and standard deviations, were calculated for the principal study variables. All items were positively worded; therefore, no reverse coding was required. For each scale, item responses were summed and divided by the number of items to obtain a mean scale score ranging from 1 to 5, with higher scores indicating higher levels of the corresponding construct. Pearson correlation coefficients were calculated to examine bivariate associations among the study variables.

#### 4.6.1. Assessment of Statistical Assumptions

Prior to the regression‐based hypothesis testing, the assumptions underlying the analyses were assessed. Normality was evaluated using skewness and kurtosis statistics, which ranged from −0.86 to 0.98 for skewness and from −1.70 to 1.38 for kurtosis, indicating approximately normal distributions of the study variables. Linearity and homoscedasticity were assessed through visual inspection of standardized residual scatterplots, with no substantial deviations observed. Independence of errors was assessed using the Durbin–Watson statistic, which was 1.73. Multicollinearity was evaluated using tolerance and variance inflation factor (VIF) statistics. Tolerance values ranged from 0.423 to 0.964, and VIF values ranged from 1.12 to 1.97, indicating no evidence of problematic multicollinearity.

#### 4.6.2. Assessment of ICU‐Level Clustering

Because participants were recruited from multiple ICUs within 10 hospitals, potential clustering at the ICU level was assessed before conducting the primary regression analyses. Random‐intercept models were used to estimate the proportion of variance attributable to differences between ICUs for integral leadership, job crafting, digital competence, and improvisational behavior. The corresponding intraclass correlation coefficients (ICCs) ranged from 0.000 to 0.011, indicating negligible between‐ICU clustering. Accordingly, the primary analyses were conducted at the individual participant level.

#### 4.6.3. Measurement Model and Confirmatory Factor Analysis (CFA)

A series of CFAs was conducted using AMOS Version 22 to evaluate the hypothesized measurement structures of the study instruments. Integral leadership, job crafting, and digital competence were specified as second‐order constructs comprising four, five, and two first‐order dimensions, respectively, whereas improvisational behavior was specified as a single‐factor construct. Standardized factor loadings ≥ 0.50 were considered acceptable, with values ≥ 0.70 indicating strong item–factor relationships [45]. Standardized item loadings ranged from 0.68 to 0.97 for integral leadership, 0.67 to 0.89 for job crafting, 0.67 to 0.85 for digital competence, and 0.75 to 0.85 for improvisational behavior (Supporting Figures S1–S4). Overall, the CFA results indicated acceptable model fit for all four instruments (Table 1).

**TABLE 1:** Confirmatory factor analysis fit indices for study instruments.

Instrument	*χ* ^2^/df	GFI	CFI	IFI	RMSEA	*p*
Integral Leadership Scale^a^	2.66	0.980	0.970	0.970	0.063	< 0.001
Clinical Nurses’ Job Crafting Scale^a^	2.92	0.930	0.940	0.930	0.067	< 0.001
Digital Competence Questionnaire^a^	2.95	0.950	0.940	0.965	0.068	< 0.001
Individual Improvisation Scale^b^	2.98	0.930	0.940	0.940	0.068	< 0.001

*Note: N* = 425. *χ* ^2^/df, chi‐square/degrees of freedom ratio.

Abbreviations: CFI, comparative fit index; GFI, goodness‐of‐fit index; IFI, incremental fit index; RMSEA, root‐mean‐square error of approximation.

^a^second‐order confirmatory factor analysis.

^b^first‐order confirmatory factor analysis.

#### 4.6.4. Reliability and Construct Validity

As reported in Table 2, internal consistency and construct validity were assessed using composite reliability (CR), McDonald’s omega (*ω*), average variance extracted (AVE), the square root of AVE, maximum shared variance (MSV), and the heterotrait–monotrait ratio (HTMT). CR and *ω* values ranged from 0.872 to 0.951, exceeding the recommended 0.70 threshold, whereas AVE values ranged from 0.584 to 0.631, exceeding 0.50 and supporting convergent validity. For discriminant validity, AVE exceeded MSV for all constructs, and the square root of AVE exceeded the corresponding interconstruct correlations reported in Table 3. HTMT values ranged from 0.223 to 0.440, remaining below the conservative 0.85 threshold.

**TABLE 2:** Construct validity and reliability of study instruments.

Construct	AVE	√AVE	MSV	CR	*ω*	1	2	3	4
1. Integral Leadership	0.631	0.794	0.109	0.950	0.951		0.431	0.361	0.313
2. Job Crafting	0.584	0.764	0.116	0.902	0.903			0.388	0.440
3. Digital Competence	0.622	0.789	0.096	0.894	0.894				0.223
4. Improvisational Behavior	0.629	0.793	0.116	0.872	0.873				

*Note: N* = 425; values in the upper triangle represent heterotrait–monotrait (HTMT) ratios of correlations. *ω*, McDonald’s omega.

Abbreviations: √AVE, square root of average variance extracted; AVE, average variance extracted; CR, composite reliability; MSV, maximum shared variance.

**TABLE 3:** Descriptive statistics and Pearson’s correlations among the study variables.

Variables	1	2	3	4
1. Integral leadership	1			
2. Job crafting	0.332^∗∗∗^	1		
3. Digital competence	0.292^∗∗∗^	0.310^∗∗∗^	1	
4. Improvisational behavior	0.246^∗∗∗^	0.336^∗∗∗^	0.166^∗∗^	1
Mean	2.79	2.79	2.63	2.83
SD	0.45	0.44	0.52	0.52

*Note: N* = 425.

Abbreviation: SD, standard deviation.

^∗∗^ *p* < 0.01 (two‐tailed).

^∗∗∗^ *p* < 0.001 (two‐tailed).

#### 4.6.5. Joint Measurement Model and Assessment of Common‐Method Variance (CMV)

A joint four‐construct CFA was conducted to evaluate the empirical distinctiveness of the focal constructs within a single measurement model. The model retained the higher‐order structures of integral leadership, job crafting, and digital competence and the single‐factor structure of improvisational behavior, with the four constructs allowed to correlate. Standardized first‐order factor loadings ranged from 0.67 to 0.97 across the four constructs, whereas second‐order factor loadings for the multidimensional constructs ranged from 0.69 to 0.87. The joint measurement model demonstrated acceptable fit, as shown in Figure 3.

**FIGURE 3:**
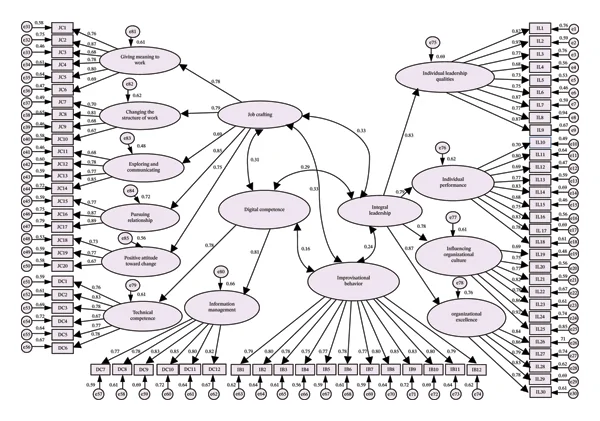
Joint confirmatory factor analysis of the four study constructs. model fit parameters: *χ* ^2^/df = 2.72, goodness‐of‐fit index (GFI) = 0.94, comparative fit index (CFI) = 0.95, incremental fit index (IFI) = 0.95, root‐mean‐square error of approximation (RMSEA) = 0.064, model chi‐square = 7096.48, DF = 2609, *p* < 0.001.

Potential CMV was assessed using two complementary approaches. First, Harman’s single‐factor test was conducted using an unrotated exploratory factor analysis of all study items. The first factor accounted for 13.487% of the total variance. Second, a common latent factor (CLF) was incorporated into the joint CFA, with paths from the CLF to all 74 observed indicators and equality constraints imposed on the unstandardized CLF paths. The baseline model (*χ* ^2^ = 7096.48, df = 2609) was compared with the CLF model (*χ* ^2^ = 7098.104, df = 2608), which demonstrated comparable fit (*χ* ^2^/df = 2.72, GFI = 0.93, CFI = 0.94, IFI = 0.94, RMSEA = 0.064). The chi‐square difference was nonsignificant (Δ*χ* ^2^ = 1.624, Δdf = 1, *p* = 0.203). Absolute changes in standardized substantive factor loadings ranged from 0.001 to 0.041, indicating that inclusion of the CLF did not materially alter the substantive measurement relationships (see Supporting Table S2).

#### 4.6.6. Moderated Mediation Analysis

The hypothesized moderated mediation model was tested using Hayes’ PROCESS macro, Model 7, with integral leadership as the independent variable, job crafting as the mediator, improvisational behavior as the dependent variable, and digital competence as the moderator, consistent with the proposed conceptual model. Analyses were adjusted for age, sex, years of experience, educational qualification, and AI use. Continuous variables involved in the interaction term were mean‐centered before analysis.

The model estimated the direct, indirect, and interaction associations using unstandardized regression coefficients (B) and 95% CIs. Conditional indirect associations were evaluated at specified levels of digital competence. Bias‐corrected bootstrap CIs based on 5000 resamples were used to evaluate indirect and conditional indirect associations; effects were considered statistically significant when the 95% bootstrap CI did not include zero. The index of moderated mediation was examined to determine whether the indirect association of integral leadership on improvisational behavior through job crafting varied as a function of digital competence. Moderated mediation was considered statistically significant when the 95% bootstrap CI for the index of moderated mediation did not include zero.

#### 4.6.7. Sensitivity Analysis

To assess the robustness of the primary findings to the exclusion of potentially influential observations, the three excluded observations (Cases 99, 243, and 365) were reintroduced, resulting in an analytical sample of 428 nurses. The complete moderated mediation analysis was repeated using the same model specification, covariates, centering procedure, and 5000‐bootstrap procedure as in the primary analysis. The sensitivity analysis showed that the individual regression paths and the integral leadership × digital competence interaction remained statistically significant. The conditional indirect association was statistically significant at the mean and high levels of digital competence but not at the low level. However, the index of moderated mediation was no longer statistically significant when the three observations were retained (index = 0.0475, 95% bootstrap CI [‐0.0061, 0.0982]), compared with a statistically significant index in the primary analysis (index = 0.035, 95% bootstrap CI [0.008, 0.071]). Thus, the individual regression relationships remained stable, whereas the overall inference regarding moderated mediation was sensitive to the inclusion of the three potentially influential observations (see Supporting Table S3).

## 5. Results

### 5.1. Sample Characteristics

The study included 425 nurses. As presented in Table 4, participants had a mean age of 37.03 years (SD = 5.59), with the majority aged between 33 and 43 years (72.9%). Female nurses represented 68.7% of the sample. More than half of the participants held a bachelor’s degree (58.1%). Nurses reported an average professional experience of 10.70 years (SD = 5.93). Additionally, 53.2% of the participants reported using AI technologies in their professional practice.

**TABLE 4:** Characteristics of the study participants.

Variable	Category	*N*	%
Age, years	Mean ± SD	37.03 ± 5.59	
	21–32	52	12.2
	33–43	310	72.9
	44–54	63	14.8

Gender	Male	133	31.3
	Female	292	68.7

Educational qualification	High technical diploma	112	26.4
	Bachelor’s degree	247	58.1
	Master’s or PhD	66	15.5

Professional experience, years	Mean ± SD	10.70 ± 5.93	
	1–5	97	22.8
	6–10	125	29.4
	11–15	74	17.4
	16–21	129	30.4

AI use	No	199	46.8
	Yes	226	53.2

*Note: N* = 425; categorical variables are presented as *n* (%); continuous variables are presented as mean ± SD.

Abbreviations: AI, artificial intelligence; SD, standard deviation.

### 5.2. Descriptive Statistics and Correlations

As shown in Table 3, the mean scores were 2.79 (SD = 0.45) for integral leadership, 2.79 (SD = 0.44) for job crafting, 2.63 (SD = 0.52) for digital competence, and 2.83 (SD = 0.52) for improvisational behavior. Pearson correlation analysis revealed statistically significant positive associations among integral leadership and job crafting (*r* = 0.332), digital competence (*r* = 0.292), and improvisational behavior (*r* = 0.246). Job crafting was positively correlated with improvisational behavior (*r* = 0.336) and digital competence (*r* = 0.310). Furthermore, digital competence demonstrated a positive relationship with improvisational behavior (*r* = 0.166).

### 5.3. Total, Direct, and Moderation Associations

As shown in Table 5, integral leadership was significantly and positively associated with improvisational behavior (*B* = 0.148, 95% CI [0.057, 0.239]). This significant total association provides support for Hypothesis 1. Table 6 presents the findings of the two regression models after controlling for age, sex, years of experience, educational qualification, and AI use. In Model 1, integral leadership was significantly and positively associated with job crafting (*B* = 0.198), while digital competence was also positively associated with job crafting (*B* = 0.151), supporting Hypotheses 2 and 4. The interaction between integral leadership and digital competence was significantly associated with job crafting (*B* = 0.205), supporting Hypothesis 5. The interaction term was associated with a significant increase in explained variance in job crafting (Δ*R* ^2^ = 0.015), indicating that the positive association between integral leadership and job crafting was stronger at higher levels of digital competence.

**TABLE 5:** Total association between integral leadership and improvisational behavior, controlling for covariates.

Predictor	*B*	SE	*Β*	*t*	*p*	95% CI
Constant	1.128	0.195	—	5.798	< 0.001	0.745, 1.510
Age	0.020	0.004	0.218	5.397	< 0.001	0.013, 0.028
Gender	−0.011	0.044	−0.010	−0.248	0.804	−0.098, 0.076
Professional experience, years	0.035	0.004	0.402	9.711	< 0.001	0.028, 0.042
AI use	0.130	0.041	0.126	3.159	0.002	0.049, 0.212
Bachelor’s degree	0.150	0.048	0.143	3.108	0.002	0.055, 0.245
Master’s or PhD	0.193	0.067	0.135	2.876	0.004	0.061, 0.325
Integral leadership	0.148	0.046	0.129	3.187	0.002	0.057, 0.239

*Note: N* = 425; high technical diploma, female, and no AI use were the reference categories for educational qualification, sex, and AI use, respectively. Model summary: *R* = 0.591; *R* ^2^ = 0.349; adjusted *R* ^2^ = 0.338; standard error of the estimate = 0.421; *F* (7, 417) = 31.932, *p* < 0.001. *B*, unstandardized regression coefficient; *β*, standardized regression coefficient.

Abbreviations: AI, artificial intelligence; CI, confidence interval; SE, standard error.

**TABLE 6:** Direct and moderation associations among the study variables.

Variable	Model 1: Job crafting				Model 2: Improvisational behavior			
	*B*	SE	*p*	95% CI	*B*	SE	*p*	95% CI
Constant	2.321	0.150	< 0.001	2.026, 2.617	1.147	0.198	< 0.001	0.758, 1.536
Age	0.006	0.003	0.112	−0.001, 0.012	0.019	0.004	< 0.001	0.012, 0.026
Sex	0.002	0.041	0.952	−0.078, 0.083	−0.011	0.044	0.811	−0.097, 0.076
Professional experience	0.010	0.003	0.002	0.004, 0.017	0.033	0.004	< 0.001	0.026, 0.040
Bachelor’s degree	0.097	0.045	0.031	0.009, 0.185	0.129	0.048	0.008	0.034, 0.223
Master’s/PhD	0.154	0.062	0.014	0.031, 0.275	0.163	0.067	0.015	0.032, 0.294
AI use	0.108	0.038	0.005	0.033, 0.183	0.113	0.041	0.006	0.032, 0.194
Integral leadership	0.198	0.045	< 0.001	0.110, 0.286	0.102	0.048	0.034	0.008, 0.196
Digital competence	0.151	0.040	< 0.001	0.073, 0.229	—	—	—	—
Job crafting	—	—	—	—	0.173	0.051	< 0.001	0.073, 0.272
Interaction term	0.205	0.071	0.004	0.065, 0.345	—	—	—	—

*Note: N* = 425; high technical diploma, female, and no AI use were the reference categories for educational qualification, sex, and AI use, respectively. Integral leadership and digital competence were mean centered before computing the interaction term (Integral leadership × digital competence). Model fit statistics. Model 1: *R* ^2^ = 0.240; MSE = 0.151; *F* (9, 415) = 14.532, *p* < 0.001. Interaction increment: Δ*R* ^2^ = 0.015; Δ*F*(1, 415) = 8.249, *p* = 0.004. Model 2: *R* ^2^ = 0.367; MSE = 0.173; *F* (8, 416) = 30.100, *p* < 0.001. B, unstandardized regression coefficient; *F*, *F* statistic; *R* ^2^, coefficient of determination; *t*, *t* statistic.

Abbreviations: AI, artificial intelligence; CI, confidence interval; DC, digital competence; IL, integral leadership; MSE, mean‐squared error; SE, standard error.

Simple slope analysis showed that the relationship between integral leadership and job crafting was nonsignificant at low levels of digital competence (*B* = 0.091), significant at moderate levels (*B* = 0.198), and strongest at high levels of digital competence (*B* = 0.305) (see Figure 4). Among the covariates, years of experience, educational qualification, and AI use were significantly associated with higher job crafting, whereas age and sex were not significant factors associated with job crafting. Model 1 explained 24.0% of the variance in job crafting (*R* ^2^ = 0.240, *F* (9, 415) = 14.532).

**FIGURE 4:**
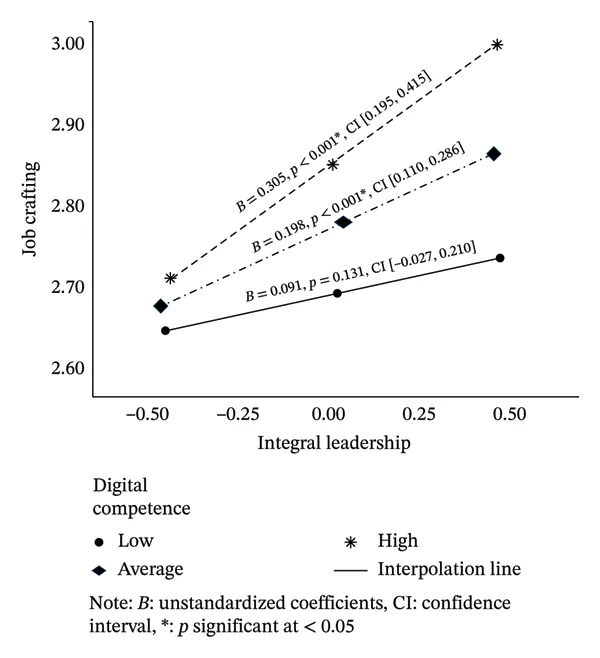
Effect of integral leadership on job crafting at levels of digital competence. Note: *B*: unstandardized coefficients, CI: confidence interval, ^∗^: *p* significant at < 0.05.

In Model 2, integral leadership was significantly associated with improvisational behavior (*B* = 0.102), which provided additional support to Hypothesis 1, while job crafting was positively associated with improvisational behavior (*B* = 0.173), supporting Hypothesis 3. Additionally, age, years of experience, educational qualification, and AI use were significantly associated with higher improvisational behavior, whereas sex was not significantly related to improvisational behavior. Model 2 accounted for 36.7% of the variance in improvisational behavior (*R* ^2^ = 0.367, *F* (8, 416) = 30.100).

### 5.4. Mediating and Moderated Indirect Associations

As presented in Table 7, an indirect association between integral leadership and improvisational behavior through job crafting was statistically significant (*B* = 0.034, SE = 0.013, 95% CI [0.011, 0.062]), supporting Hypothesis 6. The moderated mediation analysis further demonstrated that this indirect association varied significantly across levels of digital competence. The index of moderated mediation was statistically significant (*B* = 0.035, SE = 0.016, 95% CI [0.008, 0.071]), supporting Hypothesis 7.

**TABLE 7:** Moderated mediation analysis of the indirect association between integral leadership and improvisational behavior through job crafting at different levels of digital competence.

Relationship	*B*	SE	95% CI
Integral leadership ⟶ improvisational behavior	0.102	0.048	0.008, 0.196
Integral leadership ⟶ job crafting ⟶ improvisational behavior	0.034	0.013	0.011, 0.062
Conditional indirect association at low digital competence	0.016	0.012	−0.004, 0.041
Conditional indirect association at moderate digital competence	0.034	0.013	0.011, 0.062
Conditional indirect association at high digital competence	0.053	0.019	0.018, 0.092
Index of moderated mediation	0.035	0.016	0.008, 0.071

*Note: N* = 425; *B*, unstandardized coefficients.

Abbreviations: CI, confidence interval; SE, standard error.

Conditional indirect association analysis revealed that the indirect association was nonsignificant at low levels of digital competence (*B* = 0.016, 95% CI [−0.004, 0.041]). However, the indirect association became significant at moderate levels of digital competence (*B* = 0.034, 95% CI [0.011, 0.062]) and further increased at high levels of digital competence (*B* = 0.053, 95% CI [0.018, 0.092]). These findings suggest that higher digital competence was associated with a stronger indirect association between integral leadership and improvisational behavior through job crafting.

## 6. Discussion

This study examined the associations of integral leadership with critical care nurses’ improvisational behavior directly and indirectly through job crafting, and whether digital competence moderated the relationship between integral leadership and job crafting. Overall, the findings supported the proposed model. Integral leadership was positively associated with improvisational behavior and job crafting; job crafting was positively associated with improvisational behavior; and digital competence was positively associated with job crafting and strengthened the association between integral leadership and job crafting. The indirect association between integral leadership and improvisational behavior through job crafting was also stronger at higher levels of digital competence. Rather than simply confirming that leadership and job crafting are associated with positive work behaviors, the findings extend the literature by positioning improvisational behavior as a distinct behavioral outcome and by identifying digital competence as a condition that may shape how leadership is translated into proactive work redesign.

The positive association between integral leadership and improvisational behavior suggests that leadership may be an important contextual factor associated with adaptive action in intensive care settings. Integral leaders may help nurses respond more confidently to uncertainty by creating a climate in which flexibility, collaboration, and responsible adaptation are supported. This finding is consistent with studies linking leadership to innovative, adaptive, and improvisational behaviors [22–25]. The present finding adds to this literature by suggesting that the relevance of leadership extends to improvisational behavior in critical care, where unexpected demands are embedded within interconnected clinical, relational, technological, and organizational conditions.

An integral leadership orientation may provide nurses with contextual support for exercising discretion and mobilizing available resources when established routines are insufficient. Importantly, this does not imply that leadership simply encourages departure from established procedures. In critical care, improvisation may be necessary when routine responses are inadequate, but its appropriateness depends on clinical judgment, professional standards, and patient‐safety considerations. Thus, the present finding concerns the occurrence of individual improvisational behavior rather than evidence that leadership produces clinically safe or superior improvisational responses.

The significant association between integral leadership and job crafting provides further insights into how leadership becomes behaviorally meaningful. Rather than remaining an abstract perception of support, leadership appears to encourage nurses to actively reshape their work. This finding aligns with previous evidence that leadership can promote job crafting among nurses and employees in complex or digitally transforming organizations [25, 35]. In critical care, this may involve adjusting task priorities, strengthening communication patterns, seeking resources, and reinterpreting demanding work as meaningful clinical contribution. Integral leadership combines relational support with systems awareness, which may create greater latitude for nurses to proactively organize their work in response to changing demands [20]. This extends the leadership–job crafting literature by highlighting the potential importance of a leadership orientation that connects individual initiative with wider work‐system conditions.

Job crafting was also positively associated with improvisational behavior, suggesting that improvisation may be related to everyday proactive work redesign. Nurses who engage in job crafting may build stronger relational networks, situational awareness, and ownership of clinical work, which may be relevant when unexpected events require responses beyond established routines. This finding is consistent with studies linking job crafting with engagement, resilience, satisfaction, evidence‐based practice competency, and adaptive outcomes [33, 34, 37, 46]. The finding therefore extends the established role of job crafting beyond predominantly motivational, well‐being, and adaptive outcomes to a form of real‐time behavioral responsiveness that may become relevant when established work routines do not adequately address emerging demands. Proactive work redesign may shape the resources, relationships, and alternative approaches available to nurses before an unexpected situation occurs, potentially influencing how they respond when routine options become insufficient. However, improvisational behavior should not be assumed to be inherently beneficial; a spontaneous response may be functional in one situation but inappropriate in another. Therefore, the observed association with job crafting does not establish that greater job crafting produces safe improvisation.

Digital competence was positively associated with job crafting and strengthened the association between integral leadership and job crafting. This finding is consistent with the JD‐R framework, in which the value of contextual job resources may depend partly on employees’ capacity to mobilize and use those resources effectively [16], and with evidence linking digital competence with clinical judgment, digital empowerment, professional well‐being, and career sustainability [2, 14, 39]. Digital competence may therefore influence whether available technological resources function as support for proactive work redesign or contribute to documentation burden and cognitive load. More importantly, the moderation finding adds an important qualification to the leadership–job crafting relationship: Supportive leadership may provide an opportunity for proactive work redesign, but nurses’ digital capability may influence how effectively that opportunity is translated into changes in work practices.

The interaction effect was modest, accounting for an additional 1.5% of the variance in job crafting (Δ*R* ^2^ = 0.015). Its importance therefore lies less in the amount of variance explained than in identifying digital competence as a boundary condition within a behavior influenced by multiple individual, relational, and organizational factors. The finding has greater explanatory than predictive significance: digital competence helps clarify when the association between leadership and job crafting may be stronger, rather than suggesting that it accounts for a substantial proportion of job crafting behavior.

The mediation analysis showed a significant indirect association between integral leadership and improvisational behavior through job crafting, while the direct association remained significant. This partial mediation suggests that job crafting represents one pathway through which integral leadership is associated with improvisational behavior, while the remaining direct association indicates that additional pathways are likely to contribute. The finding extends previous leadership–job crafting research, including research linking leadership and job crafting with innovative work behavior among nurses [17, 18], by examining improvisational behavior as a distinct downstream correlate rather than assuming that innovative and improvisational behaviors are interchangeable.

The moderated mediation analysis further showed that this indirect association increased at higher levels of digital competence. The conditional indirect effect was nonsignificant at low digital competence (*B* = 0.016, 95%) but significant at moderate (*B* = 0.034, 95%) and high levels (*B* = 0.053, 95%). Although these conditional effects were modest, their graded pattern suggests that the association between leadership and improvisational behavior through job crafting becomes more evident as digital competence increases. Digital competence may therefore function as a complementary enabling condition through which leadership resources are more readily translated into proactive work redesign, rather than as an independent or sufficient explanation of improvisational behavior.

Taken together, the findings suggest that improvisational behavior in critical care is better understood as an emergent feature of the work environment than as an isolated individual response to unexpected events. The pattern observed in this study indicates that nurses’ capacity to improvise is embedded in the ongoing relationship between the conditions under which work is performed and the ways nurses actively engage with and reshape those conditions. From this perspective, improvisation may represent a point at which established routines encounter changing clinical demands, requiring nurses to mobilize available resources and exercise situational discretion. This interpretation broadens the understanding of improvisational behavior from an individual capacity to a contextually embedded work behavior, in which the surrounding work environment may influence how nurses respond when routine approaches become insufficient.

### 6.1. Implications for Nursing Management

The findings suggest that nurse managers may view improvisational behavior as a professional capability that can potentially be supported and strengthened, rather than solely as an accidental response to crisis. Since integral leadership was directly and indirectly associated with improvisational behavior, leadership development programs could emphasize inclusiveness, systems thinking, shared decision‐making, and support for responsible flexibility. Nurse leaders may consider creating climates where critical care nurses feel supported in adapting workflows, communicating alternative solutions, and using clinical judgment when routines are insufficient, while maintaining professional and safety standards. Because job crafting mediated the leadership–improvisation relationship, managers may also consider supporting nurses’ proactive redesign of tasks, relationships, and work meaning through autonomy, interprofessional collaboration, reflective practice, and participation in workflow improvement.

At the policy level, the findings suggest that digital competence and adaptive work redesign could be considered within nursing competency frameworks, continuing professional development requirements, and critical care standards. Policies could complement protocol‐focused requirements by recognizing the potential importance of responsible adaptation in high‐acuity environments. Since digital competence strengthened the leadership–job crafting pathway, healthcare policies may consider supporting structured digital upskilling for critical care nurses and ensuring that digital transformation initiatives include workforce readiness alongside technology implementation. Policies could also support leadership preparation programs that equip nurse leaders to foster job crafting, innovation, and context‐sensitive adaptation. The effectiveness of such educational and developmental approaches should be examined in future intervention research.

For health systems, the study highlights the potential value of integrated strategies that align leadership, digital readiness, and adaptive practice. Investments in ICU technologies may have greater practical value when accompanied by appropriate digital competence and leadership support for proactive work redesign. Health systems could therefore consider combining digital infrastructure with continuous training, supportive leadership approaches, and mechanisms for frontline nurses to improve workflows. Creating feedback systems through which nurses can report workarounds, adaptive practices, and workflow barriers may support the translation of individual improvisational experiences into organizational learning. Ultimately, an integrated approach to integral leadership, job crafting, and digital competence may contribute to critical care resilience, responsiveness, and adaptive practice in complex clinical environments.

### 6.2. Limitations and Future Directions

This study has several limitations. First, its cross‐sectional design limits causal inference and prevents determination of the temporal ordering among integral leadership, job crafting, digital competence, and improvisational behavior. Although the proposed model specifies integral leadership as the antecedent and job crafting as a pathway to improvisational behavior, the observed associations may also reflect reverse or reciprocal relationships; for example, nurses who frequently engage in improvisational behavior may subsequently perceive greater opportunities for job crafting or evaluate their leaders more positively. Future longitudinal or intervention studies are needed to examine how these relationships develop over time and whether changes in leadership, job crafting, or digital competence are followed by changes in improvisational behavior.

Second, the use of self‐reported data may increase the risk of common‐method bias, although the CLF analysis did not indicate a substantial method‐related threat. Future studies could incorporate multiple data sources, such as supervisor assessments, peer ratings, or objective indicators of adaptive performance, to provide a more comprehensive assessment of the study constructs.

Third, the participating ICUs were located in only two Egyptian governorates and were specifically selected because they had relatively advanced digital systems. This may limit the applicability of the findings to ICUs with different levels of digital infrastructure, stages of digital transformation, or organizational contexts. In addition, selecting relatively digitally advanced ICUs may have limited variation in the digital environments represented in the sample and may consequently have narrowed the range of digital competence observed among participating nurses. This selection consideration should therefore be taken into account when interpreting the observed role of digital competence in the proposed model. Future research should examine the proposed relationships across ICUs with different levels of digital infrastructure and stages of digital transformation.

Fourth, the study focused on critical care nurses, which strengthens its contextual relevance but may limit generalizability to other nursing specialties, hospitals, and healthcare systems. Future studies should replicate the model across different clinical settings, regions, and healthcare systems to determine whether the observed relationships are consistent across populations and organizational contexts.

Finally, the measurement of improvisational behavior warrants consideration when interpreting the findings. The IIS was originally developed in an entrepreneurial context, and its application in the present study represents a transfer of the measure to the critical care nursing context. The scale assesses the occurrence of individual improvisational behavior but does not capture its clinical appropriateness, safety, quality, or consequences. Therefore, greater improvisational behavior should not be interpreted as inherently desirable in critical care. Future research should distinguish the occurrence of improvisation from its clinical appropriateness and safety using healthcare‐specific measures and clinical or observational indicators.

Future research could also examine additional contextual and individual factors that may be relevant to improvisational behavior, such as psychological safety, workload, organizational culture, and digital infrastructure. Comparative and longitudinal studies could investigate whether these factors interact with leadership, job crafting, and digital competence over time and further clarify the conditions under which improvisational behavior emerges and contributes to adaptive responses in complex clinical environments.

## 7. Conclusion

This study found that critical care nurses’ improvisational behavior was associated with integral leadership, job crafting, and digital competence. Integral leadership was positively associated with improvisation both directly and indirectly through job crafting, while digital competence strengthened the association between integral leadership and job crafting and the corresponding indirect association with improvisational behavior. Taken together, these findings suggest that improvisational behavior in critical care is associated with both the work context and nurses’ proactive engagement with that context, with digital competence representing a complementary condition that may strengthen these relationships.

## Author Contributions

Conceptualization: Ahmed Abdelwahab Ibrahim El‐Sayed, Ibrahim Alasqah, Boshra Mostafa Younes, and Maha Gamal Ramadan Asal; methodology: Ahmed Abdelwahab Ibrahim El‐Sayed Ibrahim Alasqah, Maha Gamal Ramadan Asal, and Nada Alqarawi; investigation: Ahmed Abdelwahab Ibrahim El‐Sayed, Boshra Mostafa Younes, and Maha Gamal Ramadan Asal; data curation: Ahmed Abdelwahab Ibrahim El‐Sayed and Ibrahim Alasqah; formal analysis: Maha Gamal Ramadan Asal; visualization: Ahmed Abdelwahab Ibrahim El‐Sayed and Nada Alqarawi; supervision: Ahmed Abdelwahab Ibrahim El‐Sayed and Maha Gamal Ramadan Asal; validation, writing–original draft, and writing–review and editing: all authors.

## Funding

The researchers would like to thank the Deanship of Graduate Studies and Scientific Research at Qassim University (https://www.qu.edu.sa) for financial support (QU‐APC‐2026).

## Disclosure

All authors have read and approved the final version of the manuscript for submission.

## Ethics Statement

Ethical approval for this study was obtained from the Research Ethics Committee of the Faculty of Nursing, Aswan University, Egypt (IRB reference code: 6‐10‐2025). Administrative approvals were obtained from the participating hospitals. The study was conducted in accordance with the principles of the Declaration of Helsinki. Written informed consent was obtained from all participants prior to data collection.

## Consent

Please see the Ethics Statement.

## Conflicts of Interest

The authors declare no conflicts of interest.

## Supporting Information

Additional supporting information can be found online in the Supporting Information section.

## Supporting information


**Supporting Information** One supporting file is provided, containing additional analyses, sensitivity analyses, influence diagnostics, and supporting CFA figures supporting the main findings.

## Data Availability

The datasets developed and analyzed during the current work will be made available by the corresponding author upon reasonable request.
