# Supplementary material for: Unraveling Improvisational Behavior Among Critical Care Nurses in Relation to Integral Leadership, Job Crafting, and Digital Competence: A Moderated Mediation Analysis
Source: J Nurs Manag. 2026 Nov 6;2026:9316513. doi: 10.1155/jonm/9316513 (PMC13743801; doi:10.1155/jonm/9316513)
Supplement: Supplementary file 1 [file JONM-2026-9316513-s001.docx]

**Unraveling Improvisational Behavior Among Critical Care Nurses in Relation to Integral Leadership, Job Crafting, and Digital Competence: A Moderated Mediation Analysis**

**Supplementary data:**

**Table S1. Influence Diagnostics for the Three Excluded Observations**

| **Dependent variable** | **Case** | **Standardized residual** | **Studentized residual** | **Mahalanobis distance** | **Cook’s distance** | **Leverage** |
| --- | --- | --- | --- | --- | --- | --- |
| Job crafting (Mediator) | 365 | 0.92284 | 1.11231 | **132.07725** | **0.06224** | **0.30931** |
|  | 243 | 1.23509 | 1.43540 | **109.86166** | **0.08028** | **0.25729** |
|  | 99 | **−3.94973** | **−4.41200** | **83.79415** | **0.53591** | **0.19624** |
| Improvisational behavior (Outcome) | 365 | 2.85434 | 2.99486 | **38.13265** | **0.11311** | **0.08930** |
|  | 243 | 2.94439 | **3.07126** | **33.55189** | **0.10380** | **0.07858** |
|  | 99 | −2.99643 | **−3.09497** | **25.75997** | **0.08005** | **0.06033** |

Values exceeding the corresponding diagnostic cutoff are shown in bold. Standardized residuals (ZRE) were evaluated using |ZRE| > 3.00, and studentized residuals (SRE) using |SRE| > 3.00. Mahalanobis distance was evaluated against the χ² critical value at *p* < 0.001: χ²(9) = 27.88 for the job-crafting equation and χ²(8) = 26.12 for the improvisational-behavior equation. Cook’s distance was evaluated using *D* > 4/*N* = 4/428 = 0.00935. Leverage was evaluated using *hᵢ* > 2(*k* + 1)/*N*, where *k* is the number of predictors: 0.0467 for the job-crafting equation (*k* = 9) and 0.0421 for the improvisational-behavior equation (*k* = 8).

**Table S2: Common latent factor approach for common method bias**

| **First-order factor** | **Item** | **Standardized weight without CLF** | **Standardized weight with CLF** | **Difference** |
| --- | --- | --- | --- | --- |
| **Individual Leadership Qualities** | IL1 | 0.820 | 0.816 | -0.004 |
|  | IL2 | 0.972 | 0.950 | -0.022 |
|  | IL3 | 0.770 | 0.758 | -0.012 |
|  | IL4 | 0.681 | 0.68 | -0.001 |
|  | IL5 | 0.733 | 0.723 | -0.010 |
|  | IL6 | 0.751 | 0.739 | -0.012 |
|  | IL7 | 0.870 | 0.855 | -0.015 |
|  | IL8 | 0.774 | 0.790 | 0.016 |
|  | IL9 | 0.870 | 0.86 | -0.010 |
| **Individual Performance** | IL10 | 0.701 | 0.686 | -0.015 |
|  | IL11 | 0.800 | 0.794 | -0.006 |
|  | IL12 | 0.691 | 0.680 | -0.011 |
|  | IL13 | 0.769 | 0.756 | -0.013 |
|  | IL14 | 0.830 | 0.825 | -0.005 |
|  | IL15 | 0.680 | 0.690 | 0.010 |
|  | IL16 | 0.750 | 0.737 | -0.013 |
|  | IL17 | 0.831 | 0.826 | -0.005 |
|  | IL18 | 0.780 | 0.772 | -0.008 |
| **Influencing Organizational Culture** | IL19 | 0.690 | 0.678 | -0.012 |
|  | IL20 | 0.752 | 0.764 | 0.012 |
|  | IL21 | 0.772 | 0.763 | -0.009 |
|  | IL22 | 0.820 | 0.809 | -0.011 |
|  | IL23 | 0.780 | 0.790 | 0.010 |
|  | IL24 | 0.863 | 0.854 | -0.009 |
|  | IL25 | 0.920 | 0.910 | -0.010 |
| **Organizational Excellence** | IL26 | 0.842 | 0.826 | -0.016 |
|  | IL27 | 0.859 | 0.854 | -0.005 |
|  | IL28 | 0.760 | 0.801 | 0.041 |
|  | IL29 | 0.830 | 0.816 | -0.014 |
|  | IL30 | 0.780 | 0.775 | -0.005 |
| **Giving Meaning to Work** | JC1 | 0.759 | 0.751 | -0.008 |
|  | JC2 | 0.870 | 0.857 | -0.013 |
|  | JC3 | 0.680 | 0.690 | 0.010 |
|  | JC4 | 0.780 | 0.772 | -0.008 |
|  | JC5 | 0.799 | 0.788 | -0.011 |
|  | JC6 | 0.690 | 0.674 | -0.016 |
| **Changing the Structure of Work** | JC7 | 0.701 | 0.693 | -0.008 |
|  | JC8 | 0.810 | 0.807 | -0.003 |
|  | JC9 | 0.680 | 0.665 | -0.015 |
|  | JC10 | 0.670 | 0.664 | -0.006 |
| **Exploring and communicating** | JC11 | 0.681 | 0.670 | -0.011 |
|  | JC12 | 0.780 | 0.790 | 0.010 |
|  | JC13 | 0.770 | 0.764 | -0.006 |
|  | JC14 | 0.850 | 0.840 | -0.010 |
| **Pursuing Relationship Change** | JC15 | 0.770 | 0.756 | -0.014 |
|  | JC16 | 0.869 | 0.865 | -0.004 |
|  | JC17 | 0.890 | 0.881 | -0.009 |
| **Positive Attitude Toward Change** | JC18 | 0.731 | 0.717 | -0.014 |
|  | JC19 | 0.771 | 0.766 | -0.005 |
|  | JC20 | 0.671 | 0.662 | -0.009 |
| **Technical Competence** | DC1 | 0.760 | 0.748 | -0.012 |
|  | DC2 | 0.828 | 0.830 | 0.002 |
|  | DC3 | 0.780 | 0.773 | -0.007 |
|  | DC4 | 0.670 | 0.659 | -0.011 |
|  | DC5 | 0.772 | 0.755 | -0.017 |
|  | DC6 | 0.780 | 0.774 | -0.006 |
| **Information Management** | DC7 | 0.770 | 0.760 | -0.010 |
|  | DC8 | 0.782 | 0.766 | -0.016 |
|  | DC9 | 0.833 | 0.824 | -0.009 |
|  | DC10 | 0.850 | 0.840 | -0.010 |
|  | DC11 | 0.803 | 0.786 | -0.017 |
|  | DC12 | 0.820 | 0.815 | -0.005 |
| **Improvisational Behavior** | IB1 | 0.790 | 0.781 | -0.009 |
|  | IB2 | 0.798 | 0.787 | -0.011 |
|  | IB3 | 0.780 | 0.776 | -0.004 |
|  | IB4 | 0.750 | 0.742 | -0.008 |
|  | IB5 | 0.770 | 0.758 | -0.012 |
|  | IB6 | 0.780 | 0.764 | -0.016 |
|  | IB7 | 0.770 | 0.780 | 0.010 |
|  | IB8 | 0.802 | 0.789 | -0.013 |
|  | IB9 | 0.853 | 0.835 | -0.018 |
|  | IB10 | 0.830 | 0.824 | -0.006 |
|  | IB11 | 0.801 | 0.804 | 0.003 |
|  | IB12 | 0.790 | 0.776 | -0.014 |

CLF = common latent factor; IL = integral leadership; JC = job crafting; DC = digital competence; IB = improvisational behavior

**Table S3. Comparison of Primary and Sensitivity Analyses**

| **Effect / Parameter** | **Primary Analysis (N=425)** | **Sensitivity Analysis (N=428)** | **Interpretation** |
| --- | --- | --- | --- |
| Integral leadership → Job crafting | B = 0.198 | B = 0.2479 | Significant positive association in both analyses. |
| Digital competence → Job crafting | B = 0.151 | B = 0.1874 | Significant positive association in both analyses. |
| Integral leadership × Digital competence → Job crafting | B = 0.205 | B = 0.1780 | Significant interaction in both analyses. |
| ΔR² for interaction | 0.015 | 0.0249 | Interaction explained additional variance in job crafting. |
| Job crafting → Improvisational behavior | B = 0.173 | B = 0.2666 | Significant positive association in both analyses. |
| Integral leadership → Improvisational behavior | B = 0.102 | B = 0.1760 | Significant positive direct effect in both analyses. |
| Indirect effect at low digital competence | B = 0.016, 95% CI [−0.004, 0.041] | B = 0.0400, 95% CI [−0.0049, 0.0958] | Not significant in either analysis. |
| Indirect effect at mean digital competence | B = 0.034, 95% CI [0.011, 0.062] | B = 0.0661, 95% CI [0.0245, 0.1175] | Significant in both analyses. |
| Indirect effect at high digital competence | B = 0.053, 95% CI [0.018, 0.092] | B = 0.0922, 95% CI [0.0351, 0.1488] | Significant in both analyses. |
| Index of moderated mediation | B = 0.035, 95% CI [0.008, 0.071] | B = 0.0475, 95% CI [−0.0061, 0.0982] | Significant in the primary analysis but not in the sensitivity analysis. |

B = unstandardized regression coefficient; CI = 95% bootstrap confidence interval. Statistical significance for indirect effects and the index of moderated mediation was determined by whether the 95% CI excluded zero.

**Figures**

**Model Fit Parameters**:

X^2^/DF= 2.66, goodness-of-fit index (GFI)= 0.98, comparative fit index (CFI)=0.97, incremental fit index (IFI)=0.97, root mean square error of approximation (RMSEA)= 0.063, model chi square =1064.01, DF=400, p<0.001.

**Figure S1: Second order confirmatory factor analysis for the Integral Nursing Leadership scale**

**Model Fit Parameters**:

X^2^/DF= 2.92, goodness-of-fit index (GFI)= 0.93, comparative fit index (CFI)=0.94, incremental fit index (IFI)=0.93, root mean square error of approximation (RMSEA)= 0.067, model chi square =481.822, DF=165, p<0.001.

**Figure S2: Second order confirmatory factor analysis for the Clinical Nurses’ Job Crafting Scale**

**Model Fit Parameters**:

X^2^/DF= 2.95, goodness-of-fit index (GFI)= 0.95, comparative fit index (CFI)=0.94, incremental fit index (IFI)=0.965, root mean square error of approximation (RMSEA)= 0.068, model chi square = 144.552, DF=49, p<0.001

**Figure S3: Second order confirmatory factor analysis for the Digital Competency Scale**

**Model Fit Parameters**:

X^2^/DF= 2.98, goodness-of-fit index (GFI)= 0.93, comparative fit index (CFI)=0.94, incremental fit index (IFI)=0.94, root mean square error of approximation (RMSEA)=0.068, model chi square =160.921, DF=54, p<0.001

**Figure S4: a single-factor confirmatory factor analysis of Improvisational Behavior Scale**
